# Providing reducing power by microalgal photosynthesis: a novel perspective towards sustainable biocatalytic production of bulk chemicals exemplified for aliphatic amines

**DOI:** 10.1038/s41598-018-28755-6

**Published:** 2018-07-11

**Authors:** Jana Löwe, Arthur Siewert, Anna-Catharina Scholpp, Lutz Wobbe, Harald Gröger

**Affiliations:** 1 Chair of Organic Chemistry I, Faculty of Chemistry, Bielefeld University, Universitätsstr, 25, 33615 Bielefeld, Germany; 2 Algae Biotechnology and Bioenergy Group, Faculty of Biology, Center for Biotechnology/CeBiTec, Bielefeld University, Universitätsstr, 27, 33615 Bielefeld, Germany

## Abstract

A biotechnological process is reported, which enables an enzymatic reduction without the need for addition of an organic co-substrate for *in situ*-cofactor recycling. The process is based on merging the fields of enzymatic reductive amination with formate dehydrogenase-based *in situ*-cofactor recycling and algae biotechnology by means of the photoautotrophic microorganism *Chlamydomonas reinhardtii*, providing the needed formate *in situ* by formation from carbon dioxide, water and light. This biotransformation has been exemplified for the synthesis of various aliphatic amines known as bulk chemicals.

## Introduction

**Figure 1:**
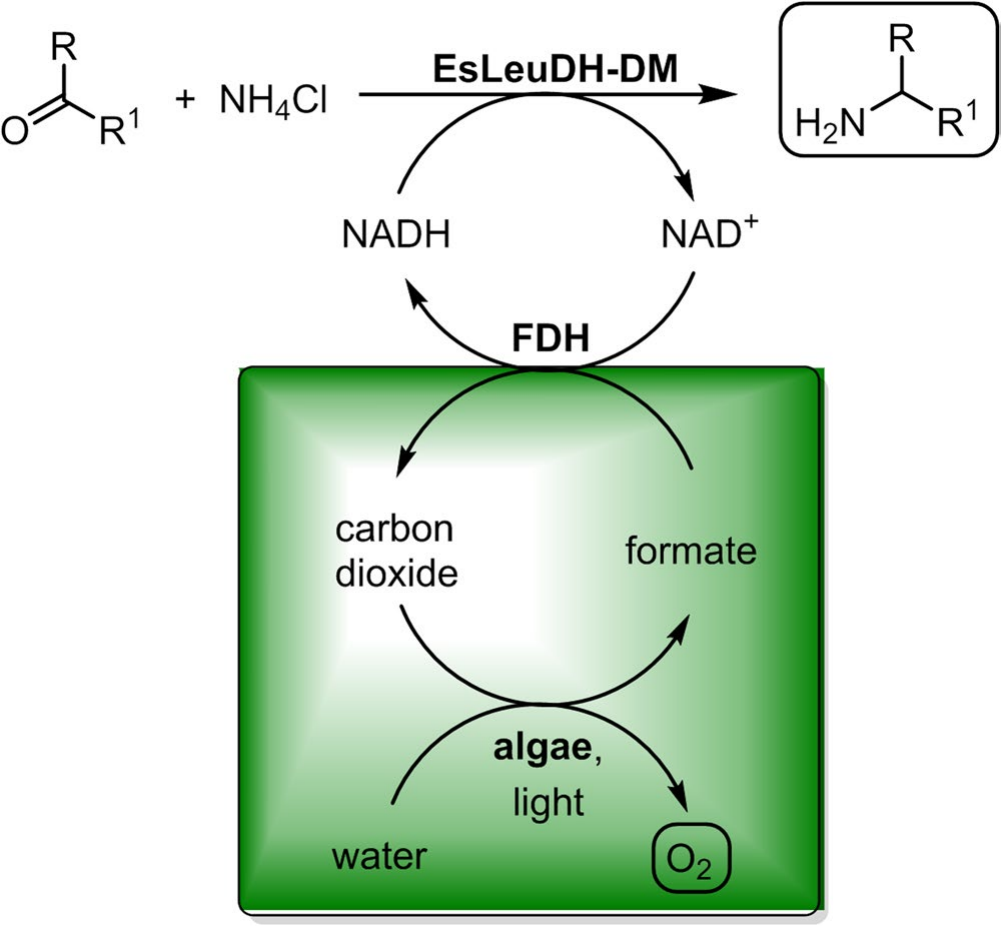
Alternative biocatalytic redox production concept for bulk amines with *in situ*-formation of the co-substrate from carbon dioxide, water and light through algae biotechnology.

Biocatalytic reduction is well established on an industrial scale, but while d-glucose or formic acid in combination with a glucose dehydrogenase and formate dehydrogenase, respectively, are highly attractive for fine chemicals production^1–3^ such organic reducing agents would represent a severe cost (and potential waste) factor in the field of bulk chemicals when being needed as a reagent added in stoichiometric amounts. Thus, for the manufacture of bulk chemicals *via* enzymatic one-step reductions identifying novel “tools” for *in situ*-cofactor recycling, which avoid the need for addition of organic co-substrates, would be desirable. In continuation of various studies with wild-type cyanobacteria^4–7^, recently Köninger *et al*. reported a promising approach relying on a recombinant form of such microorganisms, which enables a photosynthetic preparation of reducing equivalents starting from water and light and their use by a recombinant ene dehydrogenase for C=C bond reduction^8^. In the following, we present an alternative process concept based on the combination of redox enzymes with algae biotechnology according to Fig. 1, exemplified for the synthesis of bulk amines in general and hexan-1-amine in particular through enzymatic reduction. This process is based on the exclusive use of carbon dioxide, water and light for *in situ*-cofactor recycling. In detail, an microalgal cell is used which transforms carbon dioxide into formic acid, which then can be combined with a formate dehydrogenase in order to prepare the reduced cofactor form NAD(P)H (being required for the enzymatic reduction process) from the oxidized form, NAD(P)^+^. In the latter step, the carbon dioxide is formed again in the oxidation step of formic acid. In an initial step of the process, the photoautotrophic microalga *Chlamydomonas reinhardtii* reduces carbon dioxide to starch in the presence of light under aerobic conditions through photosynthesis. *Chlamydomonas* is a unicellular photosynthetic microorganism, which means that in contrast to higher plants every cell performs photosynthesis (thus avoiding “sink tissues” only respiring formed photosynthate), resulting in light-to-starch conversion efficiencies exceeding those found for higher plants (e.g. crop plants)^9^. Subsequently, in darkness and under anaerobic conditions the starch is converted into formate, which is then excreted to the reaction medium^10^. In contrast to glucose, the product of photosynthetic carbon fixation, which is kept inside the microalgal cell, fermentation products such as formate are excreted and accumulate in the culture supernatant under anaerobic conditions. The reduced cofactor NAD(P)H is needed for the enzymatic reductive amination of the carbonyl moiety, thus furnishing the desired amines and NAD(P)^+^. For this step, an amine dehydrogenase^11–14^ is used as a biocatalyst.

## Results and Discussion

### A novel concept merges microalgae biotechnology and organic biocatalysis to achieve sustainable cofactor supply

**Figure 2:**
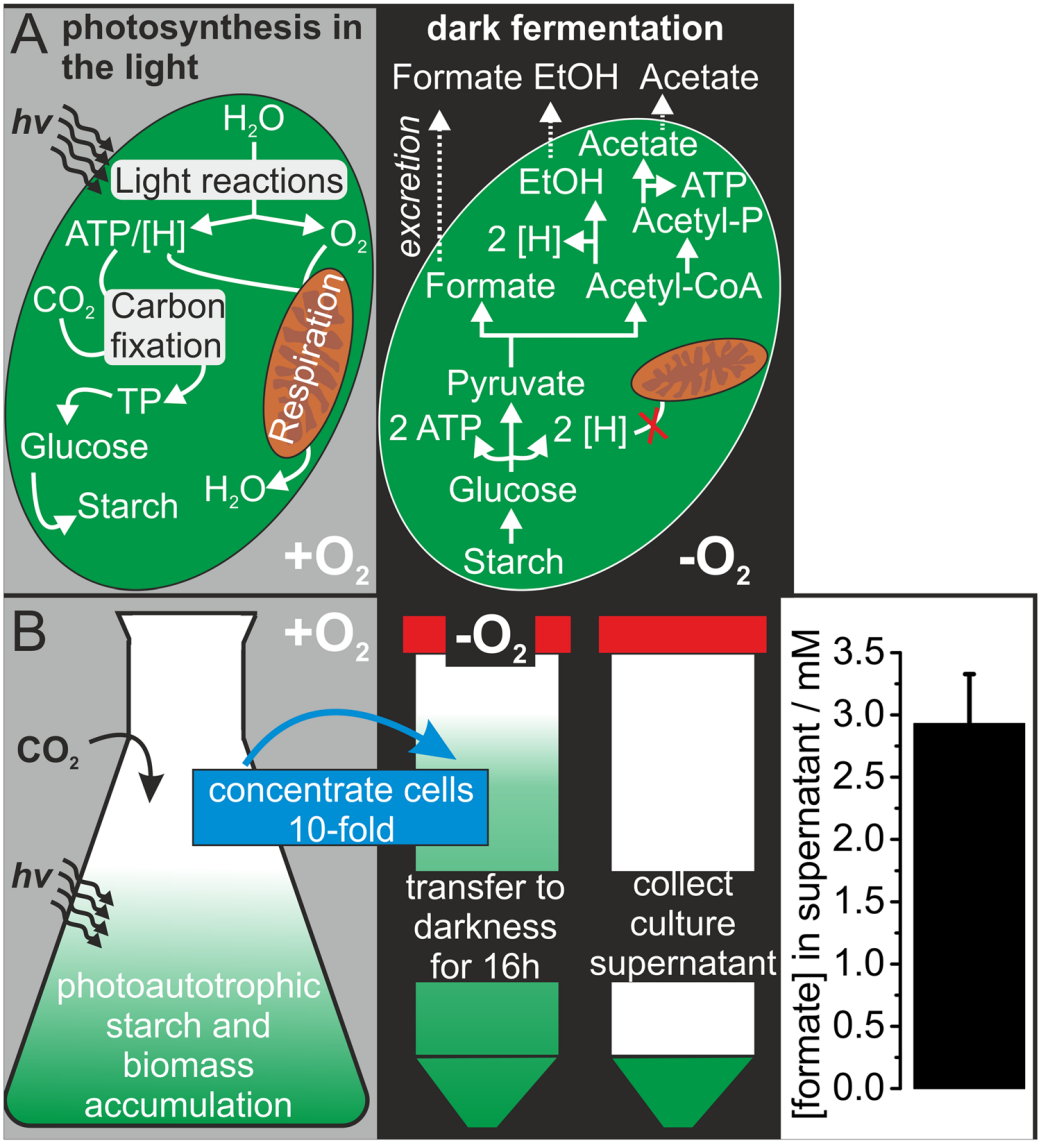
Fermentative formate production by the photoautotrophic microalga *Chlamydomonas reinhardtii*. (**A**) Left panel: Starch accumulation during the photoautotrophic phase in the presence of oxygen and light. Right panel: Starch hydrolysis, glycolytic conversion of glucose into pyruvate and fermentative production of formate from pyruvate occurring under dark anoxic conditions. (**B**) Depiction of the two-stage process applied in order to prepare formate-containing culture supernatants.

For the conversion of carbon dioxide into formate *via* photosynthetic starch production, a two-stage process was applied with *Chlamydomonas reinhardtii* as a “green photocatalyst”. This soil-dwelling microalga experiences prolonged periods of anoxic conditions, explaining why it is equipped with an extended fermentative metabolism^10^. Starch consumed by fermentation is formed during photoautotrophic growth of the microalgal cell and originates from the reduction of carbon dioxide to d-glucose within the carbon fixing reactions of photosynthesis (Fig. 2A; left panel). Reducing equivalents and ATP, required for carbon dioxide fixation, are provided by a light-driven water-splitting reaction, also leading to the formation of oxygen. In the presence of oxygen, reducing equivalents are consumed by mitochondrial respiration. A transfer of air-tight, photoautotrophic *Chlamydomonas* cultures to darkness prevents photosynthetic oxygen formation *via* water-splitting and mitochondrial respiration consumes residual oxygen until anoxic conditions are established (Fig. 2A; right panel). In the absence of light, ATP is generated by starch hydrolysis in conjunction with glycolysis^15^. Due to the absence of oxygen, reducing equivalents formed during glycolysis cannot be consumed by mitochondrial respiration and fermentative reactions sustain glycolytic flux by regenerating oxidized nicotinamide coenzymes (NAD^+^)^10^. The main fermentation products excreted by *Chlamydomonas reinhardtii* are the desired formate besides ethanol and acetate, which can be formed in a 2:1:1 ratio^15,16^ by the pyruvate formate lyase (PFL) pathway^17^. Accordingly, formate-containing algal culture supernatants were prepared in two steps, by first growing cells photoautotrophically (Fig. 2B; left panel; +O_2_) in the presence of light and CO_2_-enriched air, followed by a transfer of concentrated cell suspensions to darkness in air-tight tubes (Fig. 2B, right panel). After 16 h in darkness and separation of cells by centrifugation, formate concentrations of 2.9 ± 0.4 mM were determined in culture supernatants, which corresponds to productivities of 0.038 ± 0.003 mg formate mg^−1^ chlorophyll ·h^−1^ and was in the range of previously reported productivities (0.05 mg formate mg^−1^ chlorophyll ·h^−1^)^16^.

### Synthesis of various bulk amines via an NADH-requiring amine dehydrogenase, driven by formate oxidation in the post-fermentation supernatant of microalgal cells

After having established *in situ*-production starting from carbon dioxide, water and light, next we were interested in the combination of this microalgae-based formate production process with an enzymatic process under consumption of the *in situ*-formed formate, thus furnishing bulky amines. This enzymatic process converting readily available aldehydes into their corresponding amines was carried out by means of an amine dehydrogenase (EsLeuDH-DM), which was established by Chen *et al*. recently^14^. As target molecules we identified various aliphatic amines, which are important building blocks required on large scale by the chemicals industry^18,19^.

**Figure 3:**
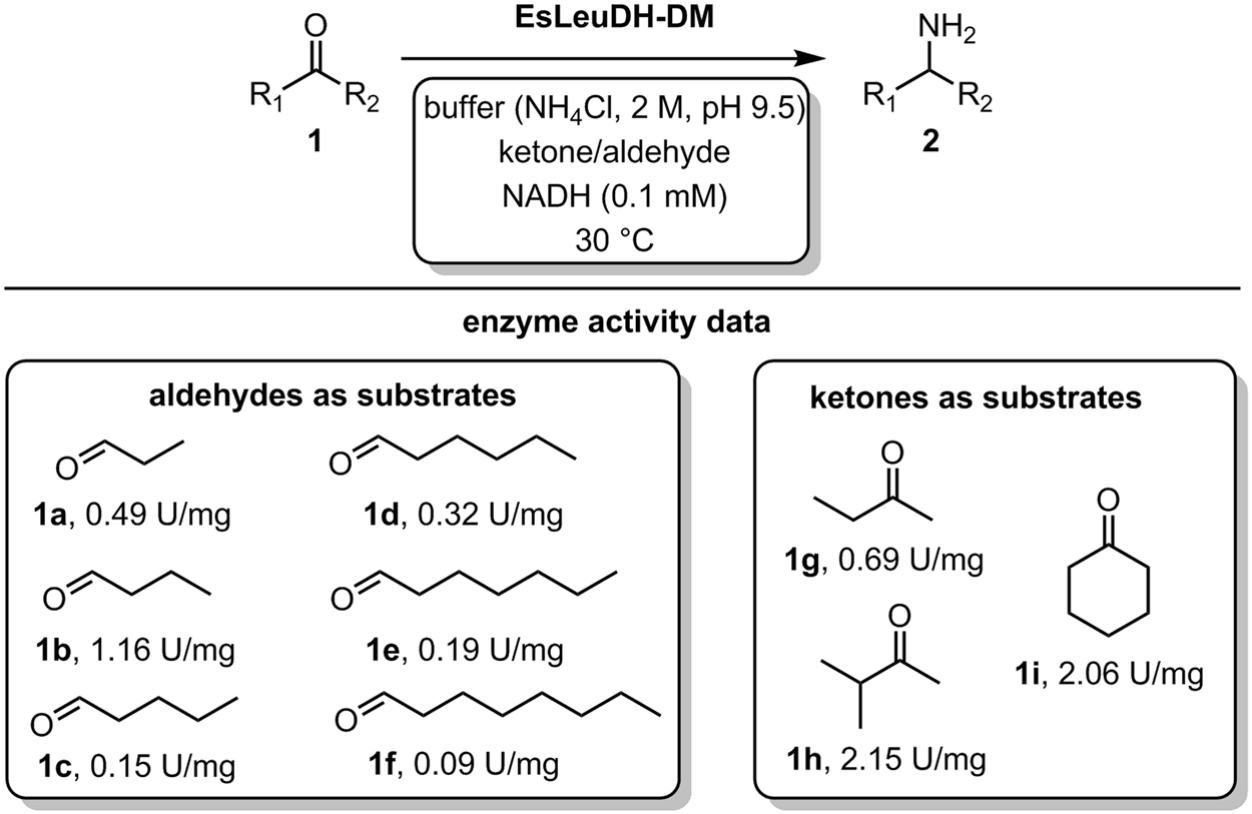
Activity of EsLeuDH-DM towards a variety of aliphatic aldehydes and ketones. Activity was measured in NH_4_Cl buffer (2M, pH 9.5) containing 0.1 mM NADH and 5 mM aldehyde or 20 mM ketone at 30 °C in a 1 mL volume.

First, the enzyme activities for these reactions were determined *via* a spectrophotometric activity assay. As substrates, short-chain and long-chain aliphatic aldehydes and short-chain ketones as well as cyclohexanone (**1i**) were chosen (Fig. 3). In 2015 Mutti *et al*. tested a range of aliphatic aldehydes with an amine dehydrogenase from *Bacillus sp*.^20^. For the amine dehydrogenase EsLeuDH-DM^14^ applied in this work, it was the first time to examine such a variety of aliphatic aldehydes for the production of bulk amines. The spectrophotometric activity assay shows synthetically useful activities for a range of substrates. The highest activity was measured for isopropyl methyl ketone (**1h**) with 2.15 U mg^−1^. In addition, EsLeuDH-DM displayed good activity towards cyclohexanone (**1i**) of 2.06 U mg^−1^. Although varying, significant enzyme activities were also found for the conversion of aliphatic aldehydes independent of the alkyl chain length. Also 2-butanone (**1g**) turned out to be a suitable substrate for the amine dehydrogenase EsLeuDH-DM. For comparison, literature values of 0.14 U mg^−1^, 2.56 U mg^−1^ and 0.87 U mg^−1^ were reported for substrates **1g**, **1h** and **1i**, respectively^14^.

**Figure 4:**
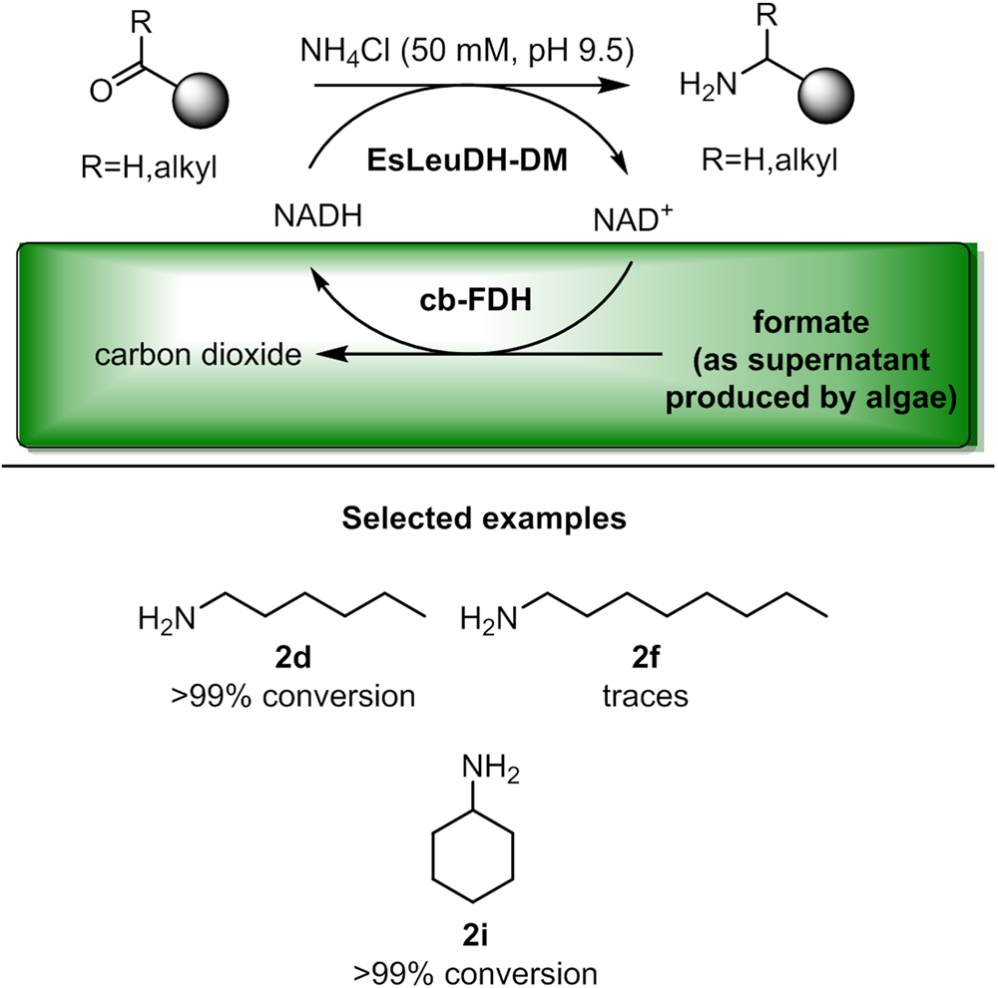
Biotransformation of EsLeuDH-DM with aliphatic carbonyl compounds as substrates (2.50 µmol) in NH_4_Cl-buffer (50 mM, pH 9.5) utilizing EsLeuDH-DM (10 U), algae supernatant (3 mM), NAD^+^ (1 µmol) and cb-FDH (3.30 U) in a total volume of 2.3 mL at 30 °C.

As the supernatant from the microalgae-based formate formation also contains acetate and ethanol (Supplementary Information; Section 6; Tables 3 and 4), their potential interference with EsLeuDH-DM activity was tested for hexanal (**1d**) as a model substrate (Supplementary Information; Section 7.2). However, there was no significant change in the activity measured. As a next step, biotransformations with this amine dehydrogenase were carried out starting from various carbonyl substrates. For *in situ*-cofactor recycling the supernatant resulting from the microalgae-catalyzed transformation of carbon dioxide and water to formate (Fig. 2) was utilized in combination with the formate dehydrogenase (FDH) from *Candida boidinii* (cb-FDH)^21^ as the required second enzyme. The photoautotrophic microorganism *Chlamydomonas reinhardtii* was used as the microalgal component described above in order to provide the needed formate in concentrations of ca. 3 mM. The process concept and experimental results are shown Fig. 4. The reaction time was set to 40 h (based on previous experience with a glucose dehydrogenase-coupled cofactor recycling, see Supplementary Information, Section 4). The reaction mixture was easily extracted with organic solvent (with no negative influence of ethanol and acetate, see Supplementary Information, Section 7.1). In general, the desired amines were formed with good to high conversion from carbonyl compounds added with a concentration of 1.1 mmol·L^−1^. For example, *n*-hexanal (**1d**) was transformed into the corresponding amine **2d** with >99% conversion, respectively. As expected from the finding that ethanol and acetate, present in microalgal supernatants after fermentation, do not perturb enzymatic amine synthesis via EsLeuDH-DM, identical results were obtained with pure formate purchased from a chemicals vendor (Supplementary Information; Section 5). Also cyclohexylamine (**2i**) was synthesized with >99% conversion in the initial experimental study. An exception represents *n*-octanal, which only led to trace amounts of octan-1-amine (**2f**). This poor conversion might be due to the low specific activity of the amine dehydrogenase for this substrate in combination with its very low solubility in aqueous reaction media.

### Bulk amines being synthesized in a one-pot reaction and in parallel to fermentative formate excretion

**Figure 5:**
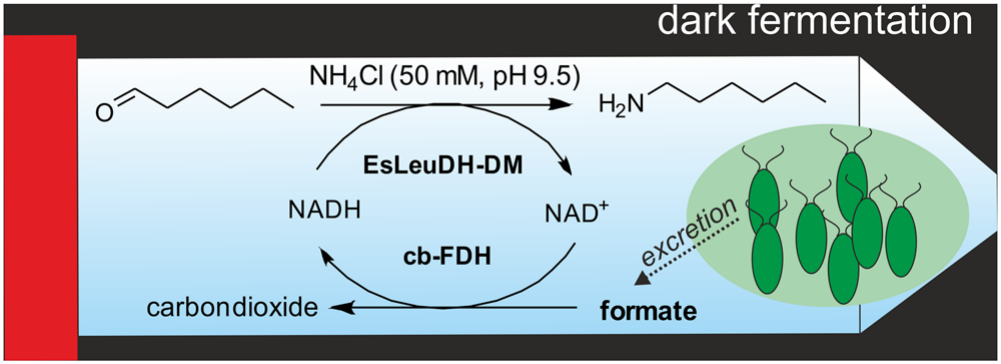
Combination of microalgae-catalyzed formate production with enzymatic amine synthesis in a one-pot process. Biotransformations were carried out with *n*-butanal (**1a**, 0.18 mg, 2.50 µmol), *n*-hexanal (**1d**, 0.25 mg, 2.50 µmol), 2-butanone (**1g**, 0.18 mg, 2.50 µmol), cyclohexanone (**1i**, 0.25 mg, 2.50 µmol) in NH_4_Cl-buffer (50 mM, pH 9.5), EsLeuDH-DM (10 U), *C. reinhardtii* cells cells (1 mL cell suspension in HSM medium; 10^8^ cells), NAD^+^ (1 µmol) and cb-FDH (3.30 U) in a total volume of 2.3 mL at 30 °C.

Next, we were interested in combining directly the microalgae-catalyzed formate production with its utilization for enzymatic amine synthesis in a one-pot process. To this end, amine dehydrogenase, formate dehydrogenase and cofactor together with the aldehyde substrate were added to *Chlamydomonas reinhardtii* cell suspensions prior to the onset of anoxia, thus enabling formate production and amine synthesis in parallel (Fig. 5). We were pleased to find that such an *in situ* formate production in the presence of an ammonia concentration of 50 mM enabled the formation of the desired butan-1-amine (**2a**), hexan-1-amine (**2d**), butan-2-amine (**2g**) and cyclohexanamine (**2i**) with excellent conversion of >99% within 40 hours (carbonyl compound concentration of 1.1 mmol·L^−1^). Negative controls, conducted for each reaction component (Supplementary Information; Section 7.3; Table 6), demonstrated that amine synthesis was strictly dependent on the formate supplied by fermenting microalgal cells.

In summary, a first proof of concept for a prospective biotechnological process has been reported, which enables an enzymatic reduction without the need for external organic co-substrate supply within *in situ*-cofactor recycling. Thus, this process concept could be in particular interesting for the area of bulk chemistry since due to the low product prices of such products the need for a co-substrate might represent a severe cost factor. Furthermore, industrial synthesis of formic acid relies on the use of fossil resources^22,23^ and will have to be replaced by other, sustainable production schemes in the future. The developed process is based on merging the fields of enzymatic reduction technologies with formate dehydrogenase-based *in situ*-cofactor recycling and algae biotechnology, providing the needed formate *in situ* by formation from carbon dioxide, water and light. This type of biotransformation has been exemplified for the use of the photoautotrophic microalga *Chlamydomonas reinhardtii* and a formate dehydrogenase for *in situ*-cofactor recycling in combination with an amine dehydrogenase to obtain aliphatic primary amines or cyclohexylamine, which all represent bulk amine products. In addition, the microalgal biomass derived from cultivation of *C. reinhardtii* is a suitable substrate for biomethane production as a cornerstone of biorefinery approaches targeting biomass valorization^23^, thus being advantageous in terms of biomass disposal issues. In spite of this advantage of an ecological *in situ*-formation of the co-substrate formate from carbon dioxide, water and light, however, limitations exist such as the low productivity of formate by microalgae. The low concentration of formate (being in the range of 3 mM) in the supernatant of fermenting *C. reinhardtii* cells currently represents the major limitation of this process concept since accordingly also the concentration of the aldehyde is limited to this concentration. Thus, work on microalgal strain development with the goal to achieve elevated formate concentrations is currently in progress.

## Methods

### Activity assay

The oxidation of NADH to NAD^+^ was measured as a decrease in absorbance at 340 nm and at 30 °C. Enzyme activities were expressed in µmol· min^−1^, using an extinction coeffizient of 6.3·10^3^ L ·mol^−1^ · cm^−1^ for NADH. The assay was performed in microtiter plates, consisting of 215 µL of 2 M ammonium chloride buffer (pH 9.5), ketone (20 mM) or aldehyde (5 mM), 15 µL NADH (10 mM, final concentration 0.15 mM) and 20 µL crude *E. coli* extract containing the enzyme (Supplementary Information; Sections 1 and 2). The activity was measured *via TecanReader*^*®*^.

### Biotransformations with microalgal supernatants

*n*-Hexanal (**1d**, 0.25 mg, 2.50 µmol), *n*-octanal (**1f**, 0.32 mg, 2.50 µmol), cyclohexanone (**1i**, 0.25 mg, 2.50 µmol) were dissolved in ammonium chloride buffer (1 mL, 100 mM, pH 9.5). EsLeuDH-DM (300 µL; 10 U), algae supernatant (1 mL, 3 mM formate) NAD^+^ (1 µL, 1 mM, 1 µmol final concentration) and commercial cb-FDH (5 mg, 3.30 U; Sigma-Aldrich F8649) were added and the mixture was heated to 30 °C. The total volume of reaction mixture was 2 mL. After 40 h the reaction was stopped. The reaction solution was extracted two times with 1 ml of MTBE (Methyl *tert*-butyl ether) per extraction step and conversion analyzed with a GC-2010 Plus from Shimadzu^®^ using the autoinjector AOC- 20i on the non-chiral Phenomenex^®^ ZB-SMS column.

### Preparation of microalgal culture supernatants enriched with formate

A *C. reinhardtii* culture grown in 1 L HSM medium (*Sueoka’s* high-salt medium)^24^ at 23 °C, bubbled with carbon dioxide-enriched air (3%; v/v) and illuminated with continuous white light (300 µmol photons m^−2^ s^−1^) was concentrated ten-fold to obtain a cell suspension in HSM medium containing ~3 mg total chlorophyll (~1.6·10^9^ cells). 25 ml of cell suspension were filled into 50 ml centrifuge tubes, wrapped with tin foil and bubbled for 5 min with nitrogen in order to remove dissolved oxygen. Air-tight tubes containing the cell suspension were then placed on an orbital shaker (200 rpm) and incubated for 16 h. Formate concentrations in cell-free culture supernatants were determined using the Formate Assay Kit (Sigma-Aldrich; MAK059).

### Biotransformations during microalgal fermentation

*n*-Butanal (**1a**, 0.18 mg, 2.50 µmol), *n*-hexanal (**1d**, 0.25 mg, 2.50 µmol), 2-butanone (**1g**, 0.18 mg, 2.50 µmol), cyclohexanone (**1i**, 0.25 mg, 2.50 µmol) were dissolved in ammonium chloride buffer (1 mL, 100 mM, pH 9.5). EsLeuDH-DM (300 µL; 10 U), photoautrophically grown *C. reinhardtii* cells (1 mL cell suspension in HSM medium; 10^8^ cells), NAD^+^ (1 µL, 1 mM, 0.5 µmol·L^−1^ final concentration) and commercial cb-FDH (5 mg, 3.30 U) were added and the mixture filled into air-tight 8 ml glass vials, wrapped with tin foil and equipped with a septum, which was used to flush the headspace with nitrogen for 5 min. The total volume of the reaction mixture was 2 mL. After 40 h of incubation at 25 °C in darkness and with constant mixing on an orbital shaker (100 rpm) the reaction was stopped. This reaction was carried out four times. The reaction solution was extracted with MTBE (2 × 1 mL). The conversion was measured with a GC-2010 Plus from Shimadzu^®^ using the autoinjector AOC- 20i on the non-chiral Phenomenex^®^ ZB-SMS column.

## Electronic supplementary material

Supplementary Information
