# Supplementary material for: Providing reducing power by microalgal photosynthesis: a novel perspective towards sustainable biocatalytic production of bulk chemicals exemplified for aliphatic amines
Source: Sci Rep. 2018 Jul 11;8:10436. doi: 10.1038/s41598-018-28755-6 (PMC6041261; doi:10.1038/s41598-018-28755-6)
Supplement: Supplementary file 1 — Supplementary Information [file 41598_2018_28755_MOESM1_ESM.pdf]

Supplementary Information

Providing reducing power by microalgal photosynthesis: a novel perspective towards sustainable biocatalytic production of bulk chemicals exemplified for aliphatic amines

Jana Löwe[1], Arthur Siewert[2], Anna-Catharina Scholpp[2], Lutz Wobbe[2]* & Harald Gröger[1]*

[1]Chair of Organic Chemistry I, Faculty of Chemistry, Bielefeld University, Universitätsstr. 25, 33615 Bielefeld, Germany; E-mail: harald.groeger@uni-bielefeld.de

[2]Algae Biotechnology and Bioenergy Group, Center for Biotechnology / CeBiTec, Bielefeld University, Universitätsstr. 27, 33615 Bielefeld, Germany

Content

1 Heterologous expression of EsLeuDH-DM in *E. coli* 2
2 Sequences 2
2.1 Leucine dehydrogenase from *Exiguobacterium sibiricum* 2
2.2 Amine dehydrogenase (EsLeuDH-DM) generated from EsLeuDH with N-terminal his-tag (used) 3
3 Calibration curve of hexanal 4
4 Kinetic experiment with glucose dehydrogenase (GDH) 5
5 Reaction with commercially available formate 6
6 Determination of ethanol and acetate amounts in *Chlamydomonas reinhardtii* supernatants during fermentation 6
7 Reaction controls 7
7.1 Extraction simulations in the presence of ethanol and acetate 7
7.2 Activity controls in the presence of ethanol and acetate 8
7.3 Negative controls for the conversion of hexanal with algal cell suspensions and algal supernatants 9
8 Chromatograms and retention times 10
9 Literature 14

1 Heterologous expression of EsLeuDH-DM in *E. coli*

TB medium (400 mL) with kanamycin (50 µg/mL) were inoculated with 1% (v/v) overnight culture. The cultures were grown at 37 °C and 180 rpm. When the EsLeuDH-DM culture reached an OD_{600nm} of 0.5, cell cultures were induced with 400 µL of IPTG (1M). For expression the temperature was reduced to 20°C.

2 Sequences

2.1 Leucine dehydrogenase from *Exiguobacterium sibiricum*

Uniprot: **B1YLR3**

Nucleotide sequence (1125 bp):

ATGGTTGAAACAAACGTAGAAGCACGATTCAGTATTTTCGAAACGATGGCAATGGAAGATTACGAACAAGT
CGTATTTTGTCACGATAAAGTCTCAGGATTAAAGGCGATTATCGCGATTCATGATACGACACTCGGACCAG
CACTCGGCGGACTCCGTATGTGGAACTATGCGTCTGACGAGGAAGCATTGATCGACGCGCTTCGTTTGGC
AAAAGGCATGACGTATAAAAATGCGGCAGCCGGTCTGAACCTTGGCGGCGGGAAAGCGGTCATCATCGGT
GATGCGAAAACGCAAAAATCAGAAGCTCTGTTCCGTGCATTCGGTCGTTACGTACAGTCGTTAAACGGACG
TTACATCACTGCGGAAGACGTCAACACAACAGTCGCCGACATGGATTATATCCACATGGAAACAGATTTCG
TAACCGGTGTCAGCCCGGCATTCGGATCAAGCGGCAATCCGTCACCAGTCACGGCTTATGGCGTTTACCG
CGGAATGAAGGCAGCCGCTAAAGAAGTATATGGCACAGATTCACTCGGAGGAAAAACAGTTGCGATTCAA
GGTGTTGGTAACGTTGCTTTCAACCTATGCCGTCACTTGCATGAAGAAGGCGCAAAATTGATTGTCACAGA
CATCAATCAAGATGCATTACGCCGTGCAGAAGAAGCGTTTGGCGCTCTCGTCGTCGGACCGGATGAAATTT
ACAGCGTCGATGCCGATATCTTTGCGCCGTGTGCCTTAGGTGCGACATTGAACGATGAGACGATTCCACAA
CTGAAAGTGAAAATCATTGCCGGAGCAGCAAACAACCAACTCAAAGAAGATCGTCACGGAGATATGCTCCA
GGAACGCGGTATTTTATATACACCGGACTTCGTCATCAACGCAGGAGGTGTCATCAATGTGGCCGACGAAC
TCGACGGGTACAACCGTGAGCGGGCGATGAAAAAAGTCGAACTCGTCTATGATGCGGTAGCAAAAGTCAT
CGAAATTGCCAAACGTGACCATCTGCCGACTTACCGGGCAGCAGAGAAGATGGCAGAAGAACGGATCGCG
ACAATGGGCAGTGCCCGCAGCCAGTTCTTACGCCGGGATAAAAACATTTTAGGATCACGCGGTTAA

Amino acid sequence (374 AS; 40,5 kDa):

MVETNVEARFSIFETMAMEDYEQVVFCHDKVSGLKAIIAIHDTTLGPALGGLRMWNYASDEEALIDALRLAKGMT
Y**K**NAAAGLNLGGGKAVIIGDAKTQKSEALFRAFGRYVQSLNGRYITAEDVNTTVADMDYIHMETDFVTGVSPAF
GSSGNPSPVTAYGVYRGMKAAAKEVYGTDSLGGKTVAIQGVGNVAFNLCRHLHEEGAKLIVTDINQDALRRAE
EAFGALVVGPDEIYSVDADIFAPCALGATLNDETIPQLKVKIIAGAA**N**NQLKEDRHGDMLQERGILYTPDFVINAG
GVINVADELDGYNRERAMKKVELVYDAVAKVIEIAKRDHLPTYRAAEKMAEERIATMGSARSQFLRRDKNILGSR
G

2.2 Amine dehydrogenase (EsLeuDH-DM) generated from EsLeuDH with N-terminal his-tag (used)

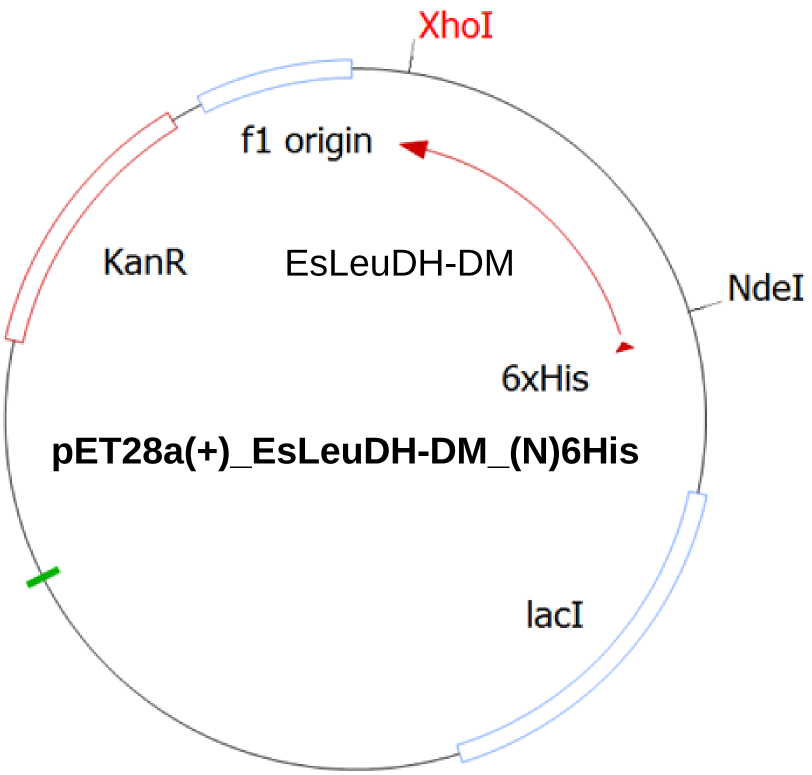

Nucleotide sequence (1185 bp):

ATGGGCAGCAGCCATCATCATCATCATCACAGCAGCGGCCTGGTGCCGCGCGGCAGCCAT
ATGGTTGAAACAAACGTAGAAGCACGATTCAGTATTTTCGAAACGATGGCAATGGAAGATTA
CGAACAAGTCGTATTTTGTCACGATAAAGTCTCAGGATTAAAGGCGATTATCGCGATTCATG
ATACGACACTCGGACCAGCACTCGGCGGACTCCGTATGTGGAACTATGCGTCTGACGAGG
AAGCATTGATCGACGCGCTTCGTTTGGCAAAAGGCATGACGTATAGCAATGCGGCAGCCG
GTCTGAACCTTGGCGGCGGGAAAGCGGTCATCATCGGTGATGCGAAAACGCAAAAATCAG
AAGCTCTGTTCCGTGCATTCGGTCGTTACGTACAGTCGTTAAACGGACGTTACATCACTGC
GGAAGACGTCAACACAACAGTCGCCGACATGGATTATATCCACATGGAAACAGATTTCGTA
ACCGGTGTCAGCCCGGCATTCGGATCAAGCGGCAATCCGTCACCAGTCACGGCTTATGGC
GTTTACCGCGGAATGAAGGCAGCCGCTAAAGAAGTATATGGCACAGATTCACTCGGAGGAA
AAACAGTTGCGATTCAAGGTGTTGGTAACGTTGCTTTCAACCTATGCCGTCACTTGCATGAA
GAAGGCGCAAAATTGATTGTCACAGACATCAATCAAGATGCATTACGCCGTGCAGAAGAAG
CGTTTGGCGCTCTCGTCGTCGGACCGGATGAAATTTACAGCGTCGATGCCGATATCTTTGC
GCCGTGTGCCTTAGGTGCGACATTGAACGATGAGACGATTCCACAACTGAAAGTGAAAATC
ATTGCCGGAGCAGCACTAAACCAACTCAAAGAAGATCGTCACGGAGATATGCTCCAGGAAC
GCGGTATTTTATATACACCGGACTTCGTCATCAACGCAGGAGGTGTCATCAATGTGGCCGA
CGAACTCGACGGGTACAACCGTGAGCGGGCGATGAAAAAAGTCGAACTCGTCTATGATGC
GGTAGCAAAAGTCATCGAAATTGCCAAACGTGACCATCTGCCGACTTACCGGGCAGCAGA
GAAGATGGCAGAAGAACGGATCGCGACAATGGGCAGTGCCCGCAGCCAGTTCTTACGCCG
GGATAAAAACATTTTAGGATCACGCGGTTAA

Amino acid sequence (394 AS; 42,7 kDa):

MGSSHHHHHHSSGLVPRGSHMVETNVEARFSIFETMAMEDYEQVVFCHDKVSGLKAIIAIHDTT
LGPALGGLRMWNYASDEEALIDALRLAKGMTYSNAAAGLNLGGGKAVIIGDAKTQKSEALFRAF
GRYVQSLNGRYITAEDVNTTVADMDYIHMETDFVTGVSPAFGSSGNPSPVTAYGVYRGMKAAA
KEVYGTDSLGGKTVAIQGVGNVAFNLCRHLHEEGAKLIVTDINQDALRRAEEAFGALVVGPDEIY
SVDADIFAPCALGATLNDETIPQLKVKIIAGAALNQLKEDRHGDMLQERGILYTPDFVINAGGVIN
VADELDGYNRERAMKKVELVYDAVAKVIEIAKRDHLPTYRAAEKMAEERIATMGSARSQFLRRD
KNILGSRG

3 Calibration curve of hexanal

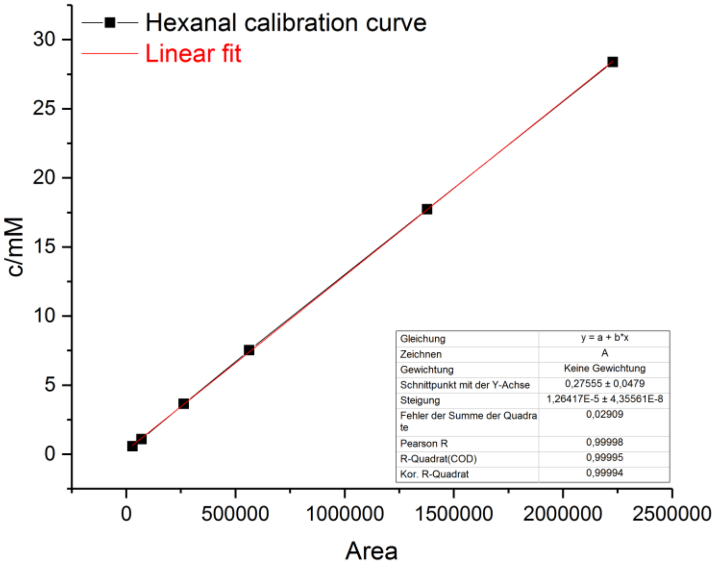

The equation for the calibration curve is shown below:

$$\text{Hexanal: } x[\text{mM}] = x \cdot 1.26 \cdot 10^{-5}\text{mM} + 0.28$$

For determination of the calibration curve hexanal (different concentrations) was dissolved in MTBE (5 mL) in a graduated measuring glass. The concentrations were measured *via* gas chromatography.

Table 1: Concentrations and areas for the calibration curve of hexanal.

Concentration/ mM	Area
0.57	28461
1.09	70234
3.64	263717
7.52	563342
17.74	1378310
28.39	2227980

4 Kinetic experiment with glucose dehydrogenase (GDH)

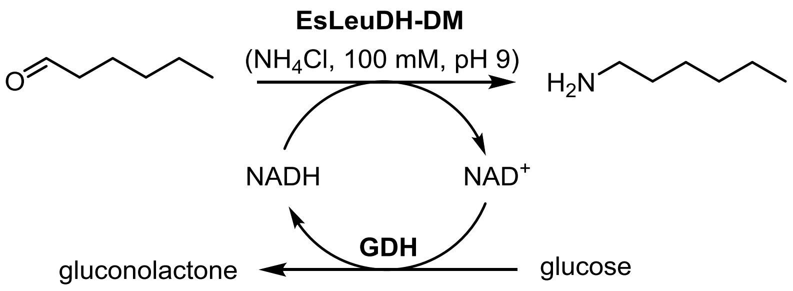

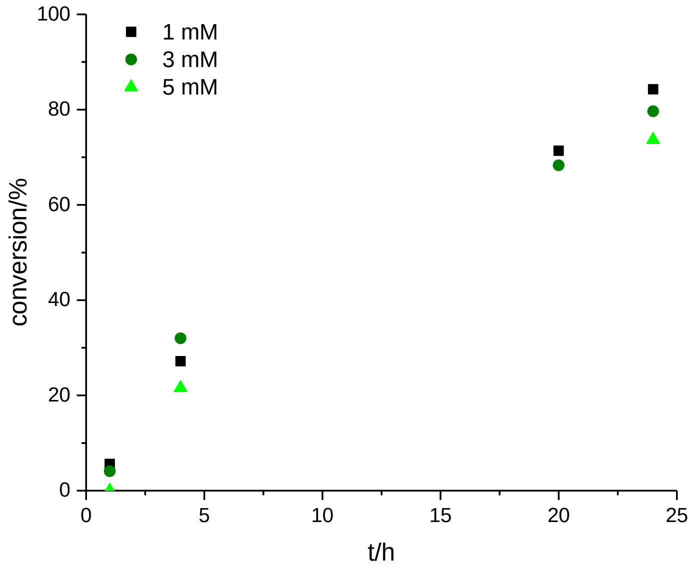

Hexanal (0.12 mg, 1.20 µmol; 0.30 mg, 3.00 µmol; 0.50, 5.00 µmol) was dissolved in ammoniumchloride buffer (0.73 mL, 2 M, pH 9.5). EsLeuDH-DM (0.30 mL; 10 U), glucose (33 µL, 1.5 M, 50 mM final concentration), NAD^+ (20 µL, 50 mM, 1 mM final concentration) and

GDH (6 µL, 42 U) were added and the mixture was heated to 30°C. At fixed times, samples were taken. The reaction solution was extracted with MTBE (2x 1 mL). The conversion was measured *via* gas chromatography.

Table 2: Results of the kinetic experiment with glucose dehydrogease for cofactor-recycling.

	Conversion/% (1h)	Conversion/% (4h)	Conversion/% (20h)	Conversion/% (24h)
**1 mM**	5.61	27.19	71.35	84.26
**3 mM**	4.09	31.98	68.30	87.51
**5 mM**	0	21.57	-	73.65

5 Reaction with commercially available formate

Hexanal (0.25 mg, 2.50 µmolI) was dissolved in ammoniumchloride buffer (1 mL, 100 mM, pH 9.5). EsLeuDH-DM (0.30 mL; 10 U), formate (69 µL, 0.1 M, 3.45 mM final concentration), NAD^+ (1 µL, 1 mM, 1 µM final concentration) and cb-FDH (5.00 mg, 3.3 U) were added and the mixture was heated to 30°C for 40 h. The reaction solution was extracted with MTBE (2 x 1 mL). The conversion was measured *via* gas chromatography. A conversion of >99% was reached.

6 Determination of ethanol and acetate amounts in *Chlamydomonas reinhardtii* supernatants during fermentation

The amount of acetate was determined according to literature[1], as well as the amount of ethanol [2]. The results for ethanol are shown in the following Table 3:

Table 3: Results of the ethanol amount in *Chlamydomonas reinhardtii*.

Sample	Extinction 1	Extinction 2	Ethanol/g L^{-1}	Ethanol/ mmol L^{-1}
**1**	0.284	1.052	0.073	1.580
**1 (diluted in H2O) 50%**	0.284	1.062	0.076	1.654
**1 (diluted in H2O) 25%**	0.276	0.960	0.048	1.041
**2**	0.285	1.033	0.067	1.465
**2 (diluted in H2O) 50%**	0.279	0.961	0.047	1.020
**2(diluted in H2O) 25%**	0.279	0.873	0.020	0.440
**3**	0.283	0.938	0.039	0.851
**3(diluted in H2O) 50%**	0.284	0.883	0.022	0.476
**3 (diluted in H2O) 25%**	0.283	0.876	0.020	0.438

For ethanol a concentration of 0.85-1.58 mmol/L was determined.

The results for acetate are shown in the following Table 4:

Table 4: Results of the acetate amount in *Chlamydomonas reinhardtii*.

Sample	Extinction 1	Extinction 2	Ethanol/g L^{-1}	Ethanol/ mmol L^{-1}
**1**	0.269	0.333	0.030	0.512
**1 (diluted in H_2O) 50%**	0.266	0.307	0.013	0.214
**1 (diluted in H_2O) 25%**	0.265	0.324	0.027	0.456
**2**	0.259	0.299	0.011	0.192
**2 (diluted in H_2O) 50%**	0.265	0.324	0.027	0.459
**2(diluted in H_2O) 25%**	0.265	0.305	0.011	0.198
**3**	0.269	0.333	0.030	0.512
**3(diluted in H_2O) 50%**	0.266	0.307	0.013	0.214
**3 (diluted in H_2O) 25%**	0.265	0.324	0.027	0.456

For acetate a concentration of 0.19-0.51 mmol/L was determined.

7 Reaction controls

7.1 Extraction simulations in the presence of ethanol and acetate

Hexanal (0.28 mg, 2.80 µmol) was dissolved in ammoniumchloride buffer (1 mL, 100 mM, pH 9.5) and in ammoniumchloride buffer (1 mL, 100 mM, pH 9.5) with ethanol (0.87 µL, 1.5 mmol), potassium acetate (0.5 mmol, 0.48 mg) and sodium formate (0.20 mg, 3 mM). The reaction mixture was heated to 30°C. The reaction solution was extracted with MTBE (2x 1 mL). The concentration was measured *via* gas chromatography and evaluated *via* calibration curve.

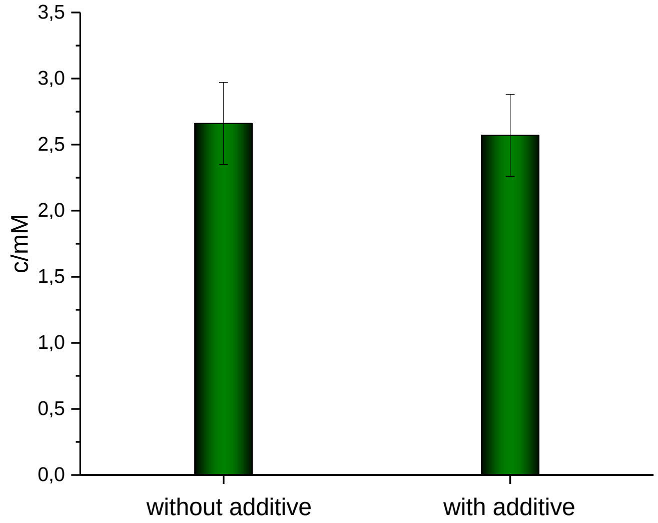

7.2 Activity controls in the presence of ethanol and acetate

The activity was measured according to the activity assay in the manuscript (see experimental section), after 0 h and 72 h incubation of enzyme, substrate and buffer, with the following composition (Table 5):

Table 5: Composition of the activity assay.

Compound	volume/µL
Buffer (NH_4Cl, pH 9.5, 100 mM, hexanal (5 mM)) or buffer (NH_4Cl, pH 9.5, 100 mM, hexanal (5 mM), ethanol (1.5 mM), potassium acetate (0.5 mM) and sodium formate (3.0 mM))	215
NADH (10 mM)	15
Enzym (crude extract)	20

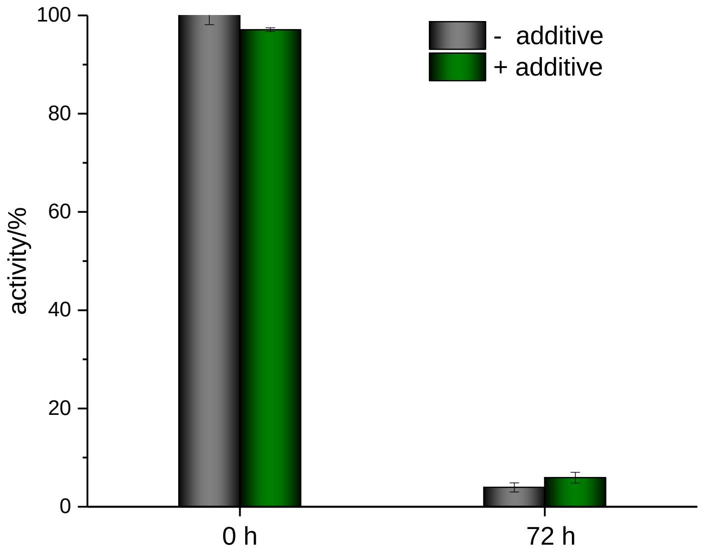

7.3 Negative controls for the conversion of hexanal with algal cell suspensions and algal supernatants

The composition of the negative controls are shown in the following (Table 6):

Table 6: Results of the negative control.

**Sample**	**NH_4Cl, pH 9.5, 100 mM, hexanal (0.25 mg, 2.5 µM)/mL**	**EsLeuDH-DM/µL**	**cb-FDH/mg**	**Cell suspension /mL**	**NAD^+ (1 mM)/µL**	**Conv./ %**
**1**	1	300	5.30	1	-	0
**2**	1		4.90	1	1	0
**3**	1	300	-	1	1	0
**4**	1					0
**5**	H_2O					0

8 Chromatograms and retention times

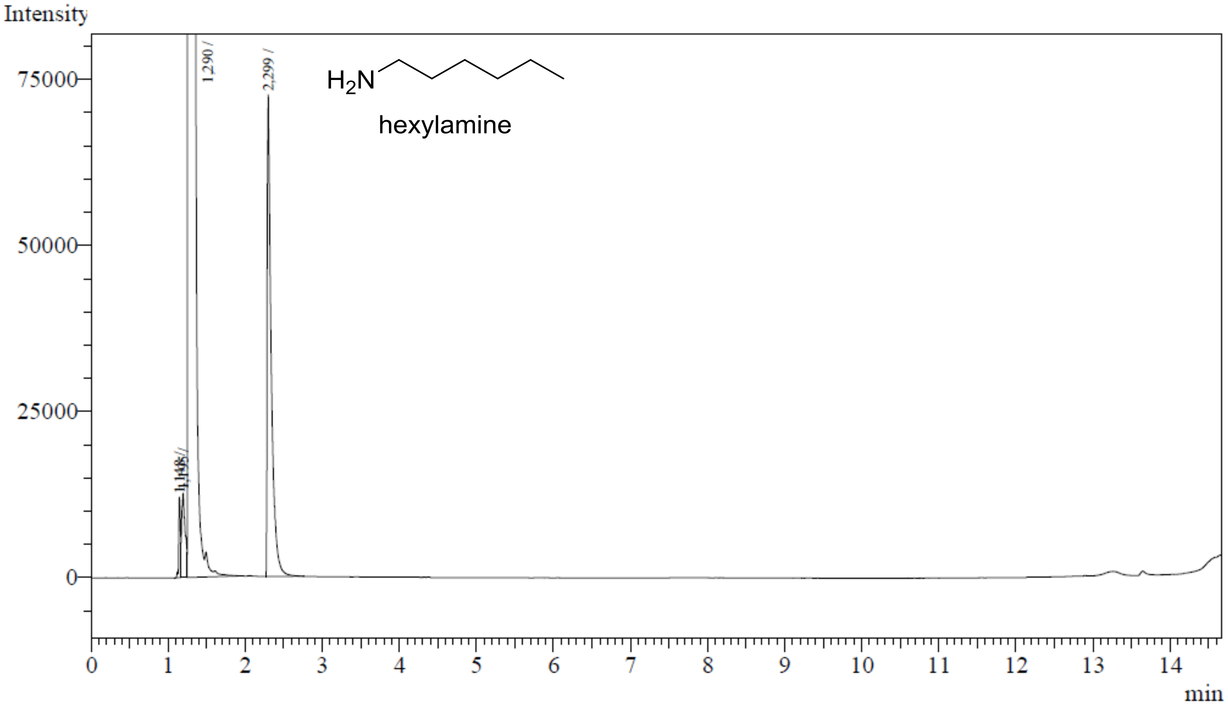

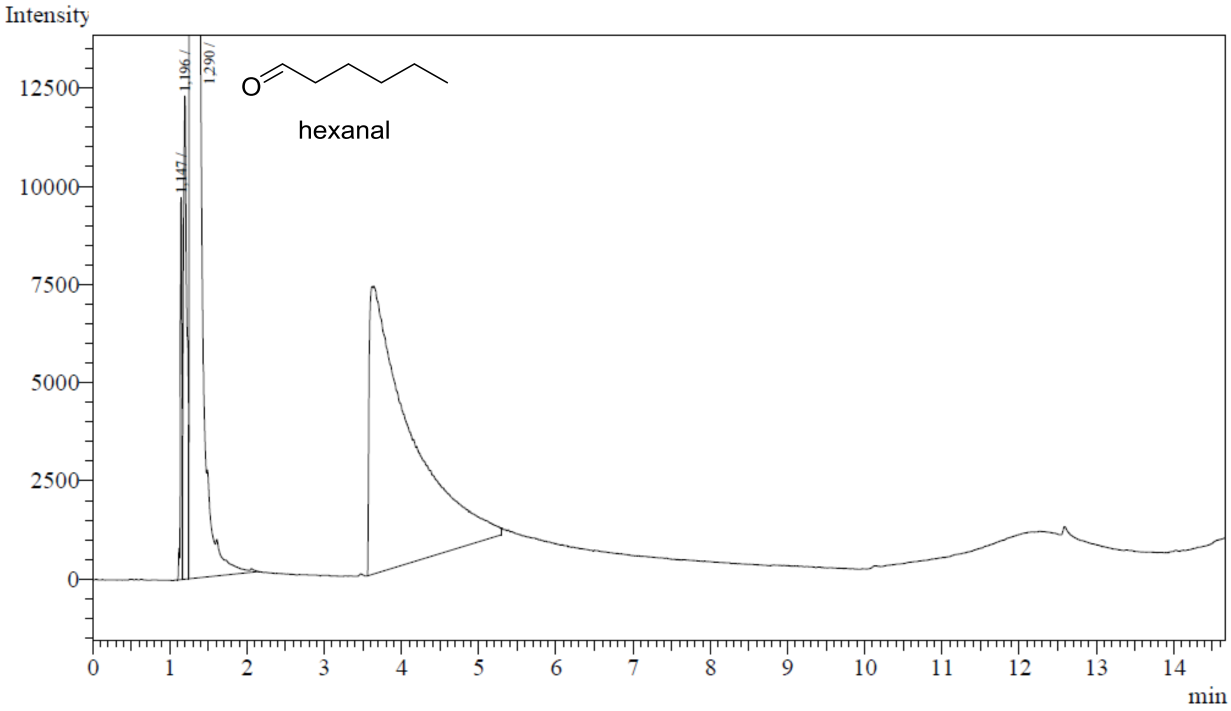

The following temperature program was used: start at 50 °C, with 3 °C/min to 80 °C and 30 °C/min to 220 °C. For hexanal (**1d**) a retention time of 2.3 min. For hexylamine (**2d**) a retention time of 3.6 min was determined.

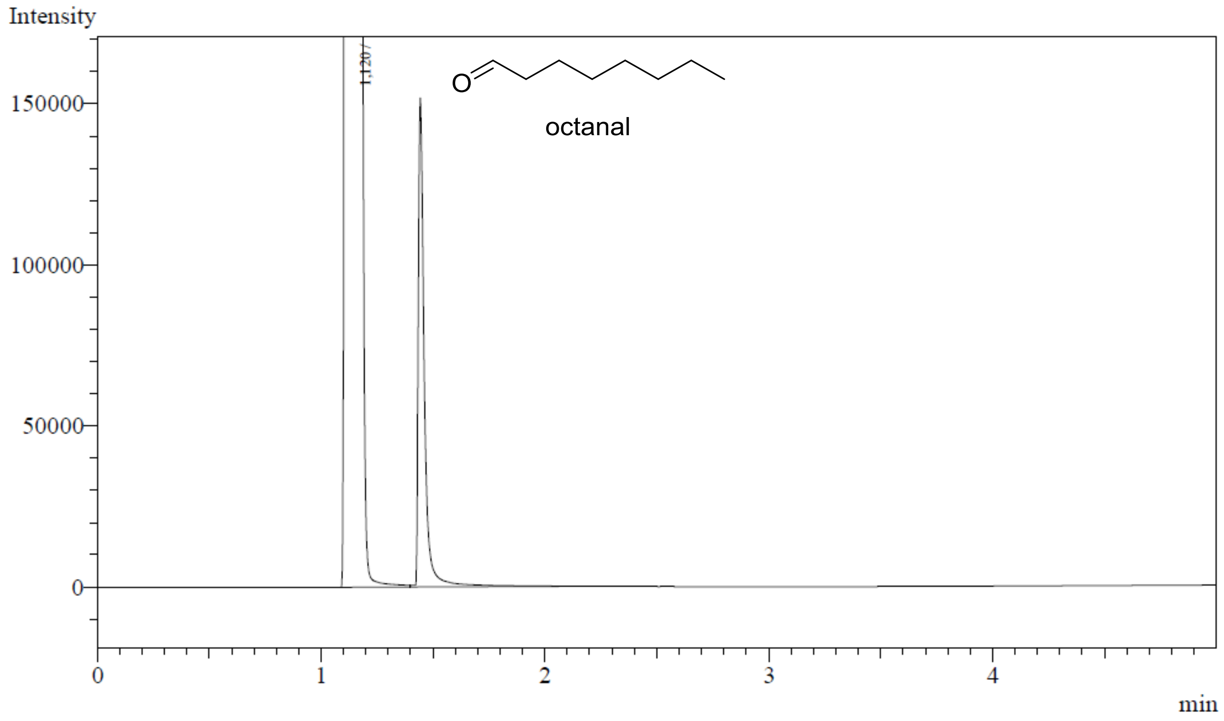

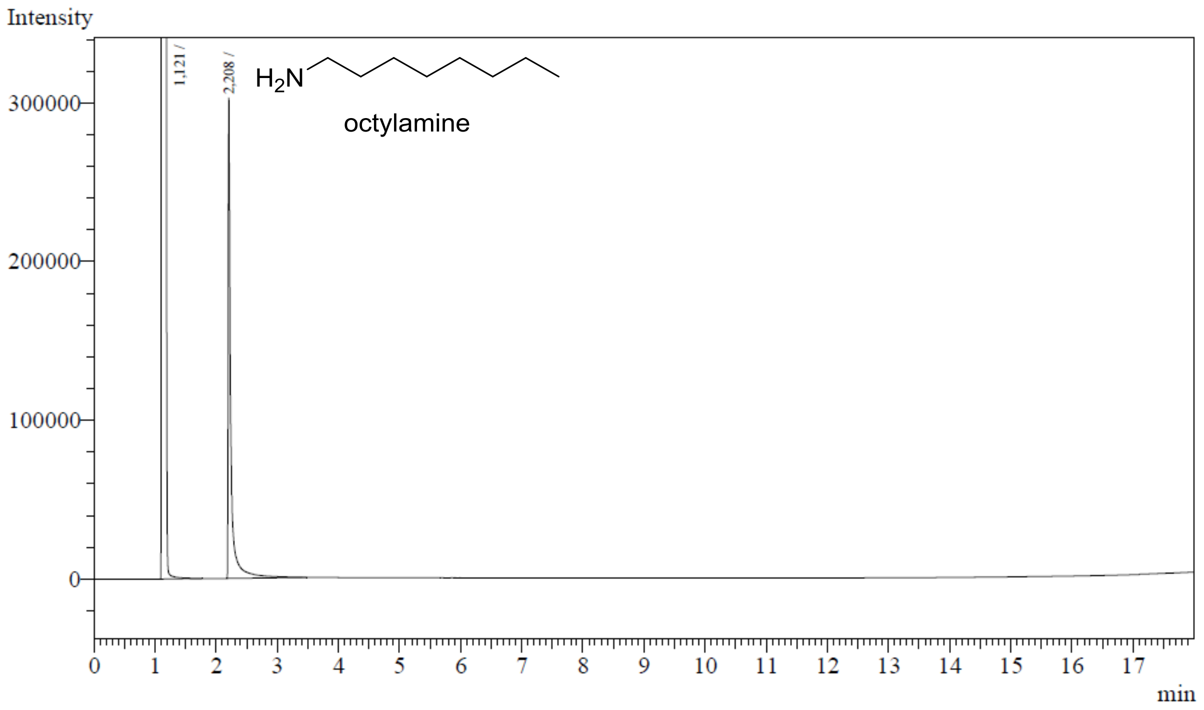

The following temperature program was used: start at 120 °C, with 10 °C/min to 300 °C. For octanal (**1f**) a retention time of 1.1 min. For octylamine (**2f**) a retention time of 2.2 min was determined.

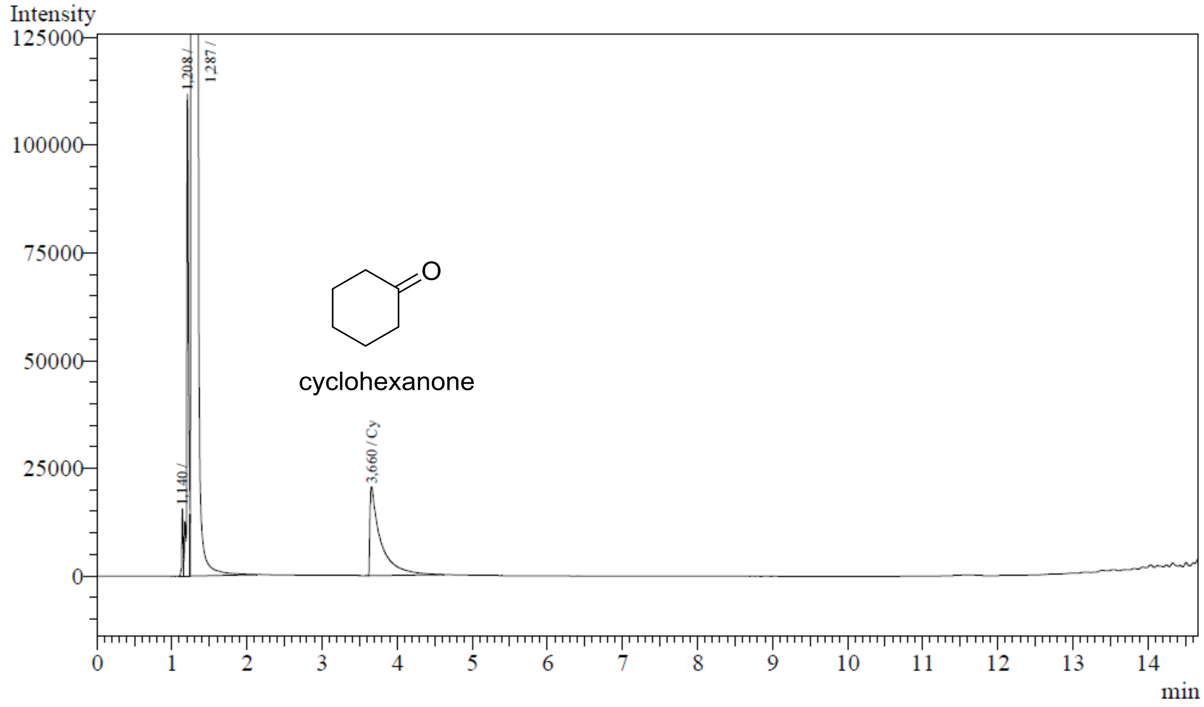

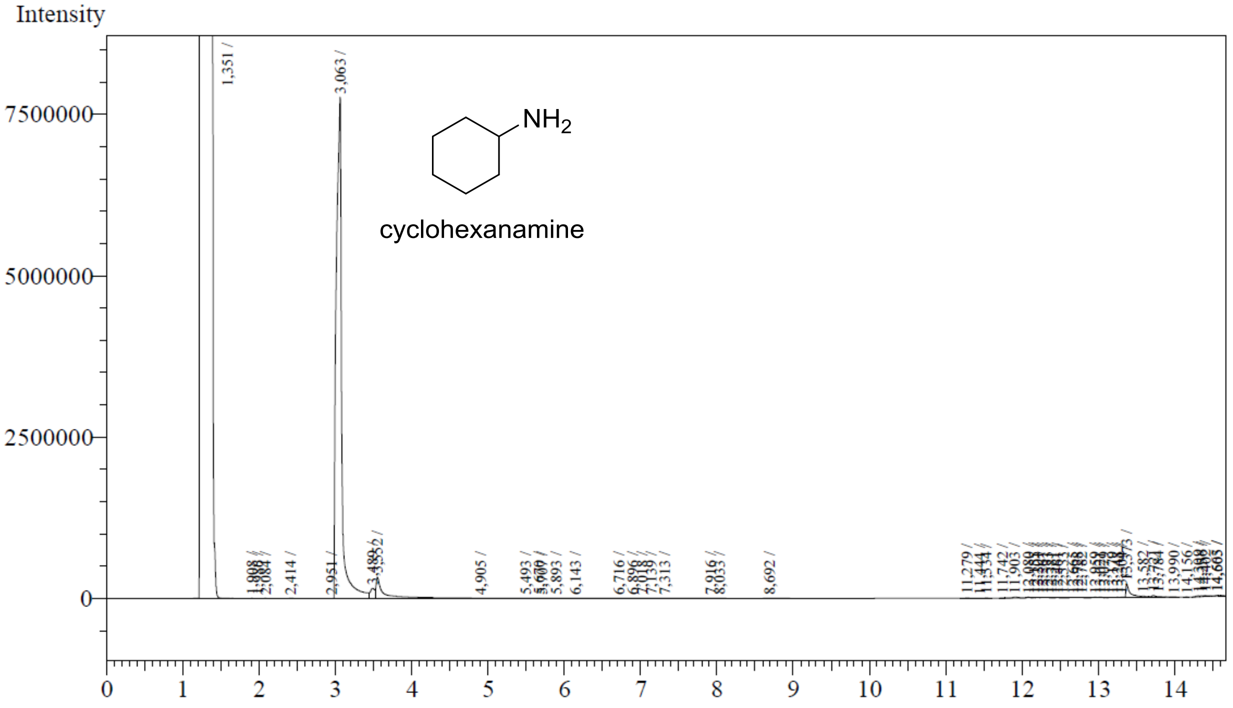

The following temperature program was used: start at 50 °C, with 3 °C/min to 80 °C. and 30 °C/min to 220 °C. For cyclohexanone (**1i**) a retention time of 3.6 min. For cyclohexylamine (**2i**) a retention time of 3.0 min was determined.

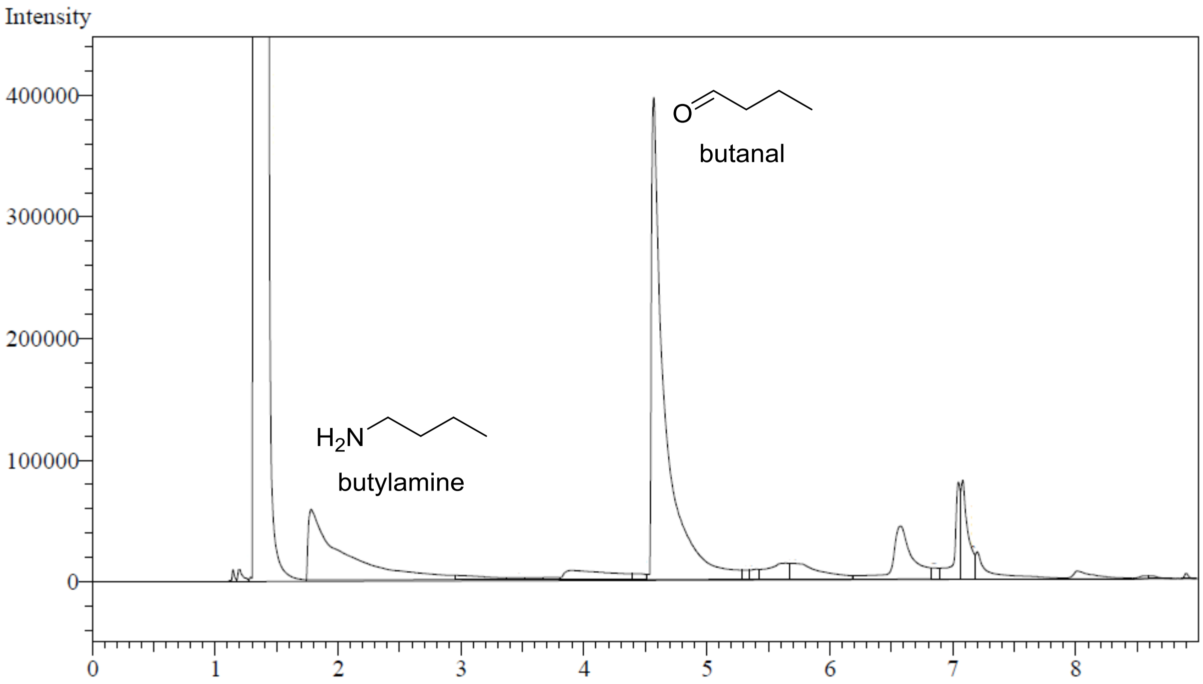

The following temperature program was used: start at 40 °C, with 3 °C/min to 50 °C. and 30 °C/min to 220 °C. For butanal (**1a**) a retention time of 1.7 min. For butylamine (**2a**) a retention time of 4.6 min was determined.

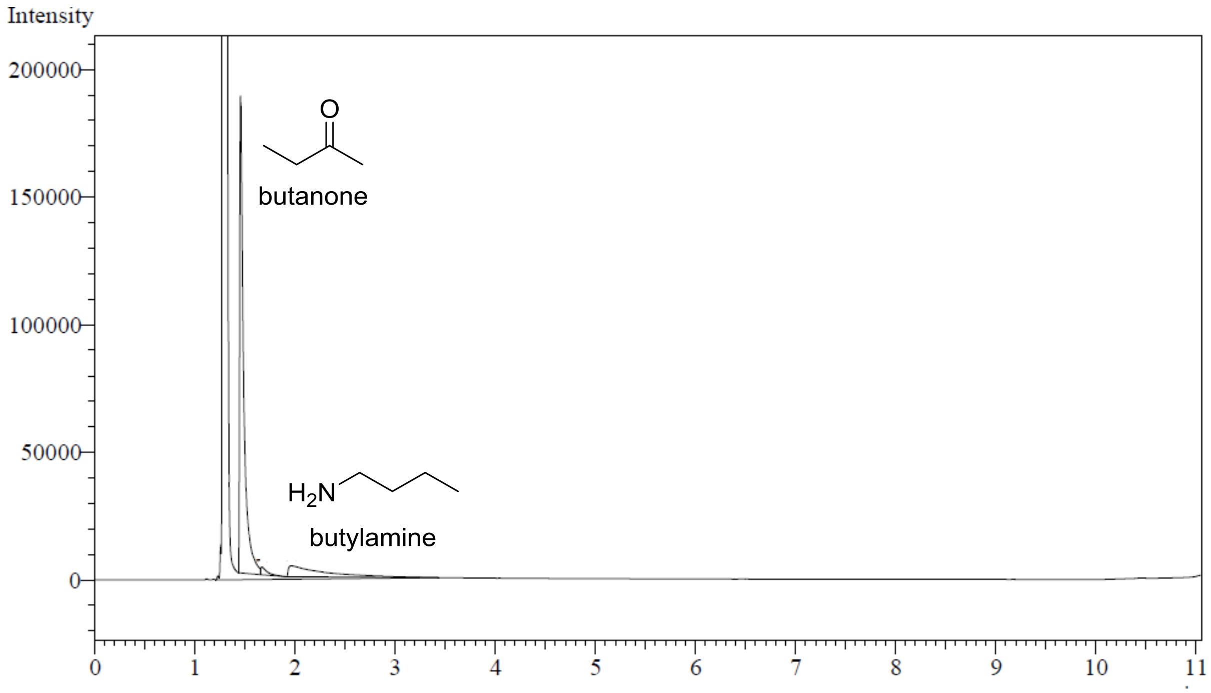

The following temperature program was used: start at 40 °C, with 3 °C/min to 50 °C. and 30 °C/min to 220 °C. For 2-butanone (**1a**) a retention time of 1.3 min. For butylamine (**2a**) a retention time of 2.0 min was determined.

9 Literature

[1] https://food.r- biopharm.com/wp- content/uploads/sites/2/2013/06/Ethanol_DE_10176290035_2013-03.pdf
[2] https://food.r- biopharm.com/wp- content/uploads/sites/2/2013/05/Acetic- acid_DE_10148261035_2013-03.pdf
